# New Unsymmetrically Benzene-Fused Bis (Tetrathiafulvalene): Synthesis, Characterization, Electrochemical Properties and Electrical Conductivity of Their Materials

**DOI:** 10.3390/ijms15034550

**Published:** 2014-03-17

**Authors:** Tahar Abbaz, Amel Bendjeddou, Abdelkrim Gouasmia, Didier Villemin, Takashi Shirahata

**Affiliations:** 1 Laboratory of Aquatic and Terrestrial Ecosystems, Organic and Bioorganic Chemistry Group, University of Mohammed Cherif Mesaadia, Souk Ahras 41000, Algeria; E-Mail: bendjeddouamel@gmail.com; 2 Laboratory of Organic Materials and Heterochemistry, University of Tebessa, Constantine Road, Tebessa 12000, Algeria; E-Mail: akgouasmia@hotmail.com; 3 Laboratory of Molecular and Thio-Organic Chemistry, UMR CNRS 6507, INC3M, FR 3038, Labex EMC3, ENSICAEN & University of Caen, Caen 14050, France; E-Mail: didier.villemin@ensicaen.fr; 4 Department of Applied Chemistry, Graduate School of Science and Engineering, Ehine University, 3 Bunkyo-cho, Matsuyama, Ehine 790-8577, Japan; E-Mail: shirahata.takashi.mj@ehime-u.ac.jp

**Keywords:** conductivity, redox potentials, organic materials, tetrathiafulvalenes

## Abstract

The synthesis of new unsymmetrically benzene-fused bis (tetrathiafulvalene) has been carried out by a cross-coupling reaction of the respective 4,5-dialkyl-1,3-dithiole- 2-selenone **6**–**9** with 2-(4-(*p*-nitrophenyl)-1,3-dithiole-2-ylidene)-1,3,5,7-tetrathia-s-indacene- 6-one **5** prepared by olefination of 4-(*p*-nitrophenyl)-1,3-dithiole-2-selenone **3** and 1,3,5,7-tetrathia-s-indacene-2,6-dione **4**. The conversion of the nitro moiety **10a**–**d** to amino **11a**–**d** then dibenzylamine **12a**–**d** groups respectively used reduction and alkylation methods. The electron donor ability of these new compounds has been measured by cyclic voltammetry (CV) technique. Charge transfer complexes with tetracyanoquino-dimethane (TCNQ) were prepared by chemical redox reactions. The complexes have been proven to give conducting materials.

## 1. Introduction

The tetrathiafulvalene (TTF) molecule has attracted great interest since the early 1970’s, when scientists saw its high electrical conductivity in a chloride salt and its metallic behaviour in the charge transfer complex, tetrathiafulvalene-tetracyanoquinodimethane (TTF-TCNQ). Recently, new applications of TTF and its derivatives in supramolecular [1–3] and materials chemistry [4–6] have been developed by TTF block building more flexible than was previously appreciated.

TTF derivatives now play a significant role as redox sites in different areas of supramolecular chemistry. Some applications call for their use as cations sensors [7–9] as a π-electron donor for non-linear optical systems [10–12], heterocycles [13–15], integrated into polymeric [16–18] and dendritic systems [19] and used as a component for molecular electronic devices [20–22].

Among the wide variety of chemical modifications performed on the TTF skeleton, the synthesis of highly extended and sulfur rich systems has recently received particular attention [23–25]. Fused aromatic rings (benzene, naphthalene, pyrazine, or quinoxaline rings) [26–28] onto the TTF skeleton are known to be an attractive electron-donor molecule which can provide a highly conductive charge transfer complex owing to its highly extended *p*-conjugate part.

As a development of our previous work [29–34] and taking into account the above, we decided to design and realize the synthesis of novel unsymmetrically benzene-fused bis (tetrathiafulvalene) containing nitrophenyl, aminophenyl or dibenzylaminophenyl units.

We report in this work the synthesis the electrochemical properties of such compounds and finally we also prepared their charge transfer complexes and measured their electrical conductivity.

## 2. Results and Discussion

**Scheme 1.**
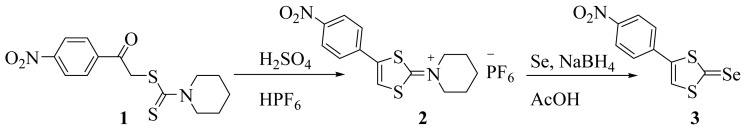
Synthetic route for the preparation of 4-(*p*-nitrophenyl)-1,3-dithiole-2-selenone **3**.

As shown in Scheme 1, commercially available 2-(*p*-nitrophenyl)-2-oxoethyl 1-piperidinecarbodithioate **1** was cyclized by the concentrated sulfuric acid at 0 °C. The resulting hydrogenosulfate was converted to 4-(*p*-nitrophenyl)-1,3-dithiole-2-ylidenepiperidinium hexafluorophosphate **2** immediately by addition of hexafluorophosphoric acid. After recrystallization, the desired product was obtained in 65% yield. The treatment of compound **2** with sodium hydrogen selenide, prepared *in situ* from selenium and sodium borohydride in ethanol at low temperature, followed by an aqueous work up, afford after filtration and purification over silica gel chromatography the desired product 4-(*p*-nitrophenyl)-1,3-dithiole-2- selenone **3** in 93% yield.

**Scheme 2.**
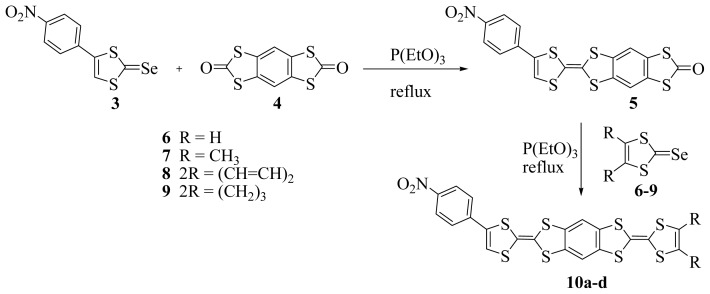
Route for the preparation of *p*-nitrophenyl benzene-fused bis tetrathiafulvalenes **10a**–**d**.

Scheme 2 exhibits the synthetic routes for the preparation of compounds **10a**–**d**. The condensation via cross coupling method [35] of The 4-(*p*-nitrophenyl)-1,3-dithiole-2-selenone **3** with 1,3,5,7-tetrathia-s-indacene- 2,6-dione **4** [28], in toluene at reflux in the presence of triethyl phosphite under nitrogen, leads to the formation of the desired 2-(4-(*p*-nitrophenyl)-1,3-dithiole-2-ylidene)-1,3,5,7-tetrathia-s-indacene- 6-one **5** in moderate yield (45%) after column chromatography. The coupling reaction between various selenones **6**–**9** [36–39] and 1,3,5,7-tetrathia-s-indacene-6-one **5** with a large excess of triethyl phosphite while refluxing in toluene successfully afforded the *p*-nitrophenyl benzene-fused bis tetrathiafulvalenes **10a**–**d** in 36%, 42%, 44% and 32% yields, respectively.

**Scheme 3.**
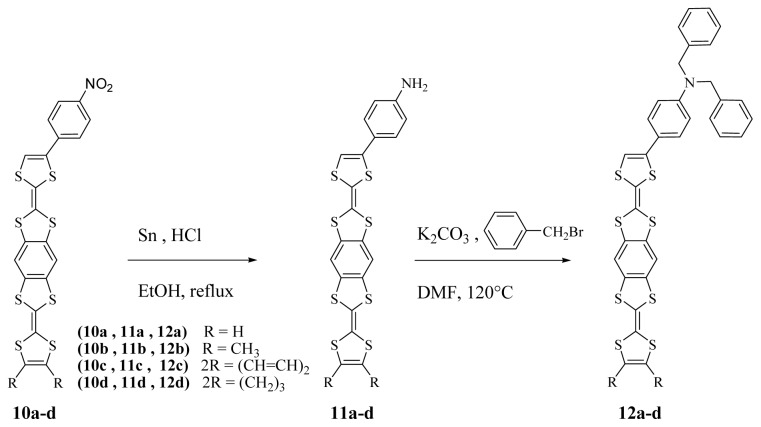
Synthetic route for the preparation of dibenzylaminophenyl benzene-fused bis

In previous work [29] we have described the access to alkylated aminophenyl bis-TTFs from nitrophenyl bis-TTFs. In Scheme 3, the nitro group of *p*-nitrophenyl benzene-fused bis tetrathiafulvalenes **10a**–**d** was reduced at reflux in the presence of tin and hydrochloric acid into an amino group in ethanol. The *p*-aminophenyl benzene-fused bis tetrathiafulvalene **11a**–**d** derivatives were obtained after purification by column chromatography in 74%, 77%, 79% and 71% yields, respectively. Then, their alkylation was effected by treatment with K_2_CO_3_ (2 equiv.) and with **2** equivalents of benzyl bromide in DMF at reflux, the dibenzylaminophenyl benzene-fused bis TTFs **12a**–**d** were obtained in 87%, 95%, 93% and 85% yields, respectively, after purification by column chromatography.

In the ^1^H NMR spectra the series of *p*-nitrophenyl benzene-fused bis tetrathiafulvalene **10a**–**d** exhibited two doublets around 7.42–7.44 and 8.08–8.10 ppm for the nitrophenyl protons. The series of *p*-aminophenyl benzene-fused bis tetrathiafulvalene **11a**–**d** revealed the presence of amino group protons signals as broad band around 3.48–3.75 ppm and the aminophenyl protons showed two doublets around 6.40–6.42 and 6.98–7.00 ppm. Thus, the series of *p*-dibenzylaminophenyl benzene-fused bis tetrathiafulvalene **12a**–**d** showed the absence of the amino group proton signals and the presence of benzylamine protons as singlet around 4.65–4.67 ppm and a multiplet around 7.13–7.28 ppm.

Mass spectrometry analysis validated the structure of the examined derivatives. In all compounds, fragmentation peaks confirmed the structure of the analyzed molecules.

### 2.1. Electrochemical Studies

The redox properties of these new functional unsymmetrically benzene-fused bis TTFs were studied in solution by cyclic voltammetry (CV) and by square wave voltammetry (SQW). Measurements were performed under nitrogen at room temperature using a glassy carbon working electrode, a Pt counter electrode and a standard calomel electrode (SCE) as reference, with tetrabutylammonium perchlorate (*n*-Bu_4_NClO_4_, 0.1 M) in dry acetonitrile, as supporting electrolyte. A scan rate of 100 mV·s^−1^ was used. The CV measurements showed reversible redox waves for all the compounds studied and the corresponding oxidation potentials *E*_ox_ were determined by the SQW technique. The results are summarized in Table 1.

**Figure 1.**
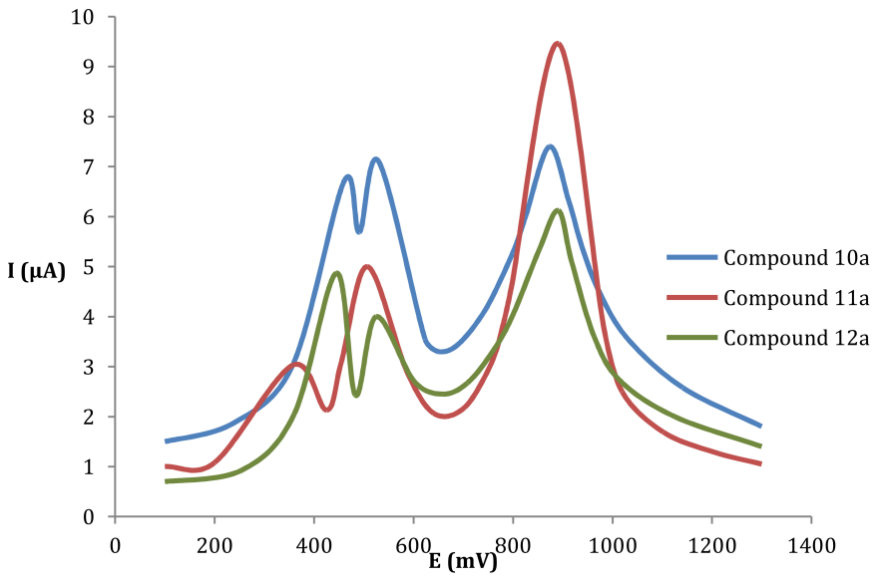
Voltammogram of benzene-fused bis TTF **10a**, **11a** and **12a**.

In Figure 1, we can clearly see three oxidation peaks with respectively a 1, 1 and 2 electron process. The real distinction of the two first oxidation waves is clearly due to the difference between the effect donor and the effect attractor of the substituents carried by the two units TTF, which also visible by cyclic voltammetry.

The oxidation potentials of compounds **12a**–**d** are slightly higher than that of compounds **11a**–**d**, on the other hand, the compounds **10a**–**d** are slightly higher than that of compounds **12a**–**d**. This should be attributable to the electron-donating capabilities of these new compounds by the presence of the *p*-nitrophenyl, *p*-aminophenyl and *p*-dibenzylaminophenyl groups linked to the donor core.

In the same series, the presence of alkyl groups on the TTF skeleton enriches the electron density and facilitates the oxidation of the donor, which it is noted for compound **10d** and **10b** compared with **10a**, while the presence of the aromatic group extends the conjugated system and improves the electron density; it was clearly visible for compound **10c** which showed the lowest oxidation potential in this series. Similar results were observed for the other series of *p*-aminophenyl and *p*-dibenzylaminophenyl groups.

### 2.2. Theoretical Calculation

The energy of HOMO of different products **10a** to **12d** was computed using DFT calculation in the Table 2. The levels of HOMO of compound **12b** (−4.507 eV) and **12d** (−4.516 eV) show that these compounds are the better donating molecule for the formation of TTF-TCNQ complexes.

**Figure 2.**
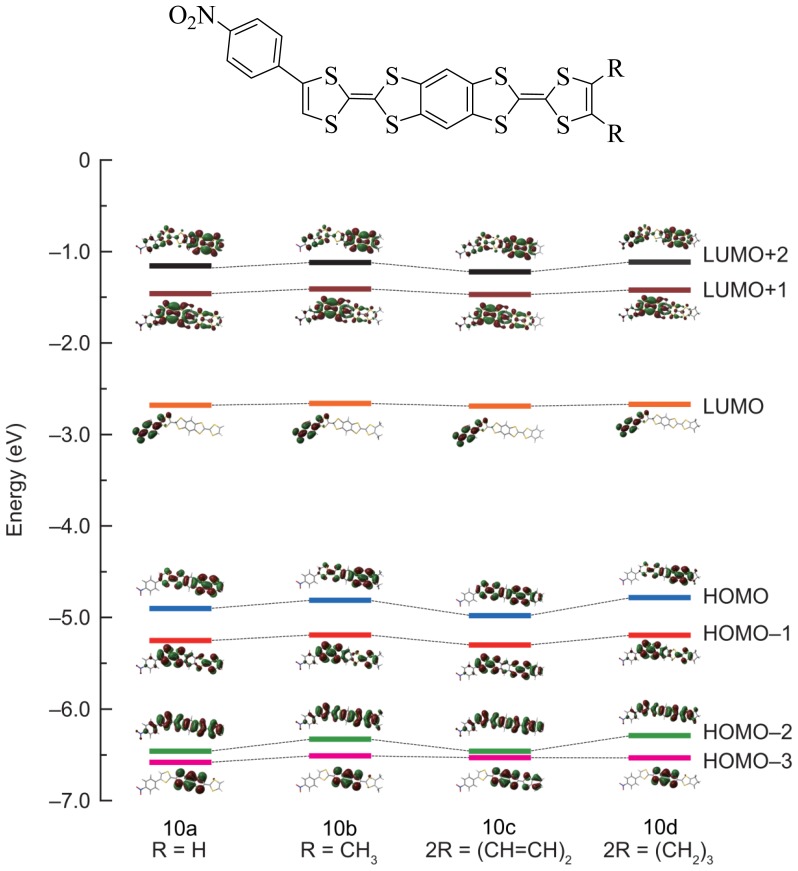
Levels of HOMO and LOMO of compounds **10a**–**d**.

Figure 2 shows that the nature of the alkyl groups has little influence on the level of the HOMO and in consequence on the potential of oxidation, which can be also found in Table 1. Three groups of compound can be obtained: **10a**–**d**, **11a**–**d** and **12a**–**d**.

**Figure 3.**
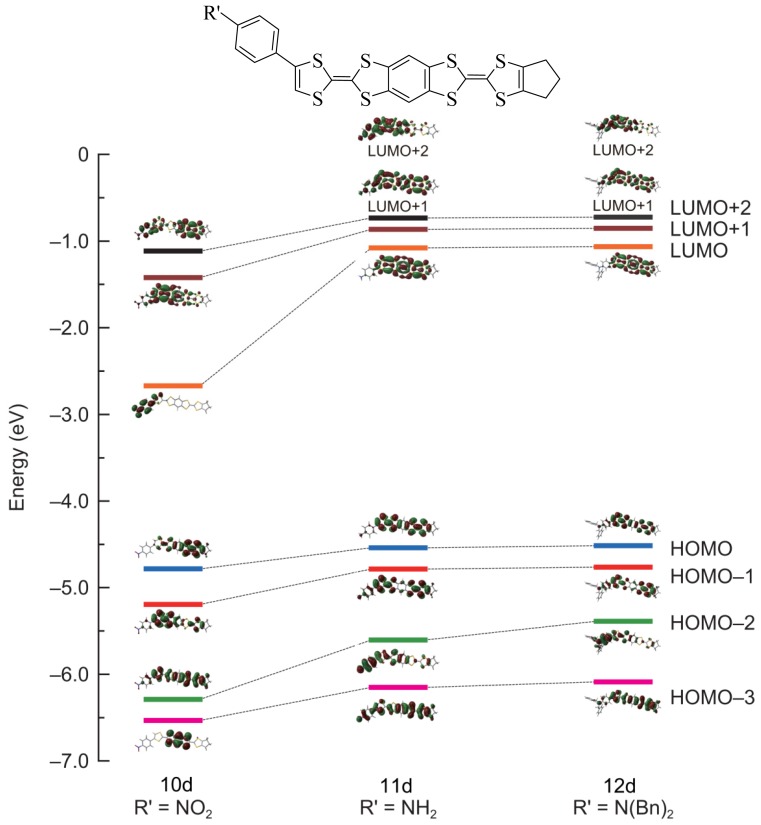
Levels of HOMO and LOMO of compounds **10d**, **11d** and **12d**.

In Figure 3 the levels of the HOMO of **10d** and **12d** shows that compound **12d** is more oxidable than **10d**, however in the case of **11d** the amine group can take part in oxidation which makes difficult a correlation between the level of the HOMO of **11d** and its facility of oxidation.

### 2.3. Preparation and Electrical Conductivity of Charge Transfer Complexes

Charge transfer complexes (CTC) are a special case where metallic-like conductivites are obtained from essentially non-metallic, organic molecules. A CTC is formed by the interaction of an electron donor (D) and an electron acceptor (A). Electron donors are compounds with low ionization potential, while electron acceptors are compounds with high electron affinity. The donor and acceptor are bound together by an electrostatic attraction, not a chemical bond. Partial electron transfer between the donor molecule and the acceptor molecule generates this electrostatic attraction.

In our study, all compounds **10a**–**12d** formed charge transfer complexes with TCNQ (tetracyano-*p*-quinodimethane) used as an electron acceptor (A) [40–42]. The solids were isolated after cooling the hot acetonitrile solution obtained by mixing equimolar amounts of the donor (D) and of TCNQ (A). Most of the materials were obtained as powders with various colors.

The room temperature conductivity of these solids was measured by using a two probe technique on compressed pellets. The results obtained are summarized in Table 3.

For this family of materials, only CTC from **10a-TCNQ** to **11d-TCNQ** resulting from *p*-nitrophenyl benzene-fused bis tetrathiafulvalenes and *p*-aminophenyl benzene-fused bis tetrathiafulvalenes, can be classified in the area of conductors. In fact, they have a conductivity measured on powder compressed pellets of 4.8 × 10^−1^ to 9.2 × 10^−2^ S cm^−1^, which allows conductivity ten times greater on single crystal.

Other, CTC resulting from *p*-dibenzylaminophenyl benzene-fused bis tetrathiafulvalenes from **12a-TCNQ** to **12d-TCNQ** can be classified in the category of semi-conductors materials with conductivities from 10^−4^ to 10^−6^ S cm^−1^. This can be due to a structural disorder and/or a full charge transfer of an electron for each molecule.

## 3. Experimental Section

### 3.1. General

NMR spectra were recorded on a WP 400-NMR instrument (Bruker BioSpin GmbH, Silberstreifen 4, 76287 Rheinstetten, Germany). FAB mass spectra were recorded on a JOEL JMS-DX 300 spectrometer (JEOL Europe, Planet II, Gebouw B., Leuvensestreenweg 542, B-1930 Zaventem, Belgium). Uncorrected melting points were measured on a 510 Buchi apparatus (BÜCHI Labortechnik AG, Meierseggstrasse 40, 9230 Flawil, Schweiz). Cyclic voltammetry measurements were carried out on a PAR-273 potentiostat/galvanostat (Alltest Instruments, Inc. 500 Central Ave. Farmingdale, NJ, USA). All computations were performed with the Gaussian 09 program package (Gaussian, Inc. 340 Quinnipiac St, Bldg 40, Wallingford, CT, USA) [43] using the 6-31G(d,p) basis set [44]. Density functional theory (DFT) calculations were carried out using a B3LYP method (public field method) [45–47]. All solvents were dried by standard methods and all commercial reagents used without purification. All reactions were performed under an inert atmosphere of nitrogen.

### 3.2. Synthesis and Characterization of 4-(p-Nitrophenyl)-1,3-dithiole-2-ylidenepiperidinium Hexafluorophosphate **2**

2-(*p*-Nitrophenyl)-2-oxoethyl 1-piperidinecarbodithioate **1** (19.44 g, 0.06 mol) was added drop wise to a stirred solution of concentrated sulfuric acid (45 mL) at 0 °C. After the reaction mixture was allowed to warm to ambient temperature, cold water (150 mL) was added and the mixture was filtered. The residual solution was cooled to 0 °C and hexafluorophosphoric acid (6.5 mL, 0.06 mol) was added drop wise over 2 min, yellow suspension was observed in the solution, and the reaction was allowed to reach room temperature. The reaction was extracted with CH_2_CI_2_ (3 × 100 mL). The organic extracts were combined and washed with water (3 × 100 mL) and dried (MgSO_4_). The solvent was removed under reduced pressure. The crude product was recrystallised from ethanol to give **2** (65%) as beige solid. M.p.: 173 °C. ^1^H NMR (400 MHz, CDCl_3_, δ, ppm): 1.78 (m, 6H, Py-*H*), 3.28 (m, 4H, Py-*H*), 7.45 (s, 1H, C*H*=C–S), 7.73 (d, *J* = 8.70 Hz, 2H, nitrophenyl-*H*), 8.17 (d, *J* = 8.70 Hz, 2H, nitrophenyl-*H*). MS (NOBA, FAB > 0): 453 [M + H]^+^. Anal. calcd. for C_14_H_15_S_2_N_2_O_2_PF_6_: C, 37.17; H, 3.34; S, 14.17; found: C, 36.87; H, 3.04; S, 14.47.

### 3.3. Synthesis and Characterization of 4-(p-Nitrophenyl)-1,3-dithiole-2-selenone **3**

Black powdered selenium (2.8 g, 35.37 mmol) was added in one portion to a solution of sodium borohydride (7.7 g, 70.74 mmol) in ethanol (40 mL) with magnetic stirring at 0 °C under argon. A vigorous reaction with considerable foaming immediately occurred and the selenium was consumed in less than 30 min. The virtually colorless solution of NaHSe, which resulted was ready for use without further treatment. After cooling of the solution acetic acid (2 mL, 35.37 mmol) and 4-*p*-nitrophenyl -1,3-dithiole-2-ylidenepiperidinium hexafluorophosphate (15.98 g, 35.37 mmol) were added and the reaction mixture was allowed to stand at room temperature for *ca.* 2 h. The ethanol was diluted to 100% with deoxygenated ice water and the red solid was filtered, washed with water, dried under vacuum and chromatographed (silica gel, CHCl_3_). Recrystallization of the product from heptane gave **3** (9.94 g, 93% yield) as red orange crystals. M.p.: 146 °C. TLC:Rf = 0.90 (CH_2_Cl_2_). ^1^H NMR (400 MHz, CDCl_3_, δ, ppm): 6.94 (s, 1H, C=C*H*), 7.57 (d, *J* = 8.65 Hz, 2H, nitrophenyl-*H*), 8.17 (d, *J* = 8.65 Hz, 2H, nitrophenyl-*H*); MS (NOBA, FAB > 0): 303 [M + H]^+^. Anal. calcd. for C_9_H_5_S_2_SeNO_2_: C, 35.76; H, 1.66; S, 21.21; found: C, 35.46; H, 1.46; S, 21.51.

### 3.4. Synthesis and Characterization of 2-(4-(p-Nitrophenyl)-1,3-dithiole-2-ylidene)-1,3,5,7-tetrathia-s-indacene- 6-one **5**

Under a nitrogen atmosphere, 25 mL of freshly distilled triethyl phosphite was added to the mixture of 4-(*p*-nitrophenyl)-1,3-dithiole-2-selenone **3** (1 g, 3.31 mmol) and 1,3,5,7-tetrathia-s-indacene-2,6- dione **4** (1 equiv.). The resulting mixture was heated with an oil bath up to 110 °C and stirred for a further 4 h. The solvent was then removed under reduced pressure. Compound **5** was obtained by column chromatography of the residue (silica gel, eluting with dichloromethane and petroleum ether 2:1) in 45% yield. Light yellow powder, M.p.: 132 °C. TLC: Rf = 0.83 (CH_2_Cl_2_). ^1^H NMR (400 MHz, CDCl_3_, δ, ppm): 6.83 (s, 1H, C=C*H*), 7.11 (s, 2H, benzene-fused-*H*), 7.45 (d, *J* = 8.87 Hz, 2H, nitrophenyl-*H*), 8.10 (d, *J* = 8.87 Hz, 2H, nitrophenyl-*H*); MS (NOBA, FAB > 0): 466 [M + H]^+^. Anal. calcd. for C_17_H_7_S_6_NO_3_: C, 43.85; H, 1.51; S, 41.31; found: C, 44.00; H, 1.71; S, 41.01.

### 3.5. Synthesis and Characterization of p-Nitrophenyl Benzene-Fused Bis Tetrathiafulvalene **10a**–**d**

Compounds **10a**–**d** were synthesized by employing the same experimental process as **5** from 1 equiv. of **5** and 1 equiv. of various selenones **6**–**9**.

*p-*Nitrophenyl benzene-fused bis tetrathiafulvalene **10a**: Dark blue powder. Yield: 36%. M.p.: 168 °C. TLC: Rf = 0.70 (CH_2_Cl_2_/petroleum ether, 2:1). ^1^H NMR (400 MHz, CDCl_3_, δ, ppm): 6.37 (s, 2H, C*H*=C*H*), 6.84 (s, 1H, C=C*H*), 6.95 (s, 2H, benzene-fused-*H*), 7.44 (d, *J* = 8.88 Hz, 2H, nitrophenyl-*H*), 8.10 (d, *J* = 8.88 Hz, 2H, nitrophenyl-*H*). MS (NOBA, FAB > 0): 552 [M + H]^+^. Anal. calcd. for C_20_H_9_S_8_NO_2_: C, 43.53; H, 1.64; S, 46.48; found: C, 43.73; H, 1.74; S, 46.18.

*p-*Nitrophenyl benzene-fused bis tetrathiafulvalene **10b**: Midnight blue powder. Yield: 42%. M.p.: 175 °C. TLC: Rf = 0.65 (CH_2_Cl_2_/petroleum ether, 2:1). ^1^H NMR (400 MHz, CDCl_3_, δ, ppm): 1.95 (s, 6H, C*H*_3_), 6.82 (s, 1H, C=C*H*), 6.91 (s, 2H, benzene-fused-*H*), 7.42 (d, *J* = 8.86 Hz, 2H, nitrophenyl-*H*), 8.08 (d, *J* = 8.86 Hz, 2H, nitrophenyl-*H*). MS (NOBA, FAB > 0): 580 [M + H]^+^. Anal. calcd. for C_22_H_13_S_8_NO_2_: C, 45.56; H, 2.25; S, 44.23; found: C, 45.86; H, 2.55; S, 43.93.

*p-*Nitrophenyl benzene-fused bis tetrathiafulvalene **10c**: Indigo powder. Yield: 44%. M.p.: 184 °C. TLC: Rf = 0.54 (CH_2_Cl_2_/petroleum ether, 2:1). ^1^H NMR (400 MHz, CDCl_3_, δ, ppm): 6.83 (s, 1H, C=C*H*), 6.93 (s, 2H, benzene-fused-*H*), 7.00–7.30 (m, 4H, benzene-*H*), 7.43 (d, *J* = 9.00 Hz, 2H, nitrophenyl-*H*), 8.10 (d, *J* = 9.00 Hz, 2H, nitrophenyl-*H*). MS (NOBA, FAB > 0): 602 [M + H]^+^. Anal. calcd. for C_24_H_11_S_8_NO_2_: C, 47.89; H, 1.84; S, 42.62; found: C, 48.09; H, 2.04; S, 42.52.

*p-*Nitrophenyl benzene-fused bis tetrathiafulvalene **10d**: Blue violet powder. Yield: 32%. M.p.: 188 °C. TLC: Rf = 0.58 (CH_2_Cl_2_/petroleum ether, 2:1). ^1^H NMR (400 MHz, CDCl_3_, δ, ppm): 2.45 (q, *J* = 6.9 Hz, 2H, C*H*_2_), 2.56 (t, *J* = 6.9 Hz, 4H, 2C*H*_2_), 6.82 (s, 1H, C=C*H*), 6.91 (s, 2H, benzene-fused-*H*), 7.42 (d, *J* = 8.87 Hz, 2H, nitrophenyl-*H*), 8.08 (d, *J* = 8.87 Hz, 2H, nitrophenyl-*H*). MS (NOBA, FAB > 0): 592 [M + H]^+^. Anal. calcd. for C_23_H_13_S_8_NO_2_: C, 46.67; H, 2.21; S, 43.34; found: C, 46.77; H, 2.31; S, 43.19.

### 3.6. Synthesis and Characterization of p-Aminophenyl Benzene-Fused Bis Tetrathiafulvalene **11a**–**d**

A stirred mixture of 4-*p*-nitrophenyl benzene-fused bis TTFs derivatives **10a**–**d** (4 mmol), tin (0.94 g, 8 mmol), and aqueous solution of HCl (35%) to (1.8 mL, 20 mmol) in ethanol (30 mL) was refluxed for 4 h under nitrogen. During this time the initial black solution turned light yellow. The solution was then concentrated *in vacuo* and treated with an aqueous solution (100 mL) of sodium hydroxide (0.1 M) and extracted with ether. The organic phase was washed with water, dried (MgSO_4_), and concentrated *in vacuo*. The product was subjected to column chromatography on silica gel (CH_2_Cl_2_), affording the expected compounds **11a**–**d** as powder.

*p-*Aminophenyl benzene-fused bis tetrathiafulvalene **11a**: Dark orange powder. Yield: 74%. M.p.: 127 °C. TLC: Rf = 0.72 (CH_2_Cl_2_). ^1^H NMR (400 MHz, CDCl_3_, δ, ppm): 3.50–3.75 (br, 2H, NH_2_), 6.37 (s, 2H, CH=C*H*), 6.42 (d, *J* = 8.48 Hz, 2H, aminophenyl-*H*), 6.60 (s, 1H, C=CH), 6.95 (s, 2H, benzene-fused-*H*), 7.00 (d, *J* = 8.48 Hz, 2H, aminophenyl-*H*); MS (NOBA, FAB > 0): 522 [M + H]^+^. Anal. calcd. for C_20_H_11_S_8_N: C, 46.03; H, 2.12; S, 49.15; found: C, 46.22; H, 2.27; S, 48.83.

*p-*Aminophenyl benzene-fused bis tetrathiafulvalene **11b**: Orange powder. Yield: 77%. M.p.: 133 °C. TLC: Rf = 0.67 (CH_2_Cl_2_). ^1^H NMR (400 MHz, CDCl_3_, δ, ppm): 1.95 (s, 6H, C*H*_3_), 3.48–3.73 (br, 2H, N*H*_2_), 6.40 (d, *J* = 8.26 Hz, 2H, aminophenyl-*H*), 6.58 (s, 1H, C=C*H*), 6.91 (s, 2H, benzene-fused-*H*), 6.98 (d, *J* = 8.26 Hz, 2H, aminophenyl-*H*). MS (NOBA, FAB > 0): 550 [M + H]^+^. Anal. calcd. for C_22_H_15_S_8_N: C, 48.05; H, 2.74; S, 46.64; found: C, 48.33; H, 2.97; S, 46.36.

*p-*Aminophenyl benzene-fused bis tetrathiafulvalene **11c**: Coral powder. Yield: 79%. M.p.: 142 °C. TLC: Rf = 0.56 (CH_2_Cl_2_). ^1^H NMR (400 MHz, CDCl_3_, δ, ppm): 3.59–3.89 (br, 2H, N*H*_2_), 6.59 (s, 1H, C=C*H*), 6.41 (d, *J* = 8.60 Hz, 2H, aminophenyl-*H*), 6.93 (s, 2H, benzene-fused-*H*), 6.99 (d, *J* = 8.60 Hz, 2H, aminophenyl-*H*), 7.00–7.30 (m, 4H, benzene-*H*). MS (NOBA, FAB > 0): 572 [M + H]^+^. Anal. calcd. for C_24_H_13_S_8_N: C, 50.40; H, 2.29; S, 44.85; found: C, 50.70; H, 2.39; S, 45.20.

*p-*Aminophenyl benzene-fused bis tetrathiafulvalene **11d**: Orange red powder. Yield: 71%. M.p.: 146 °C. TLC: Rf = 0.60 (CH_2_Cl_2_). ^1^H NMR (400 MHz, CDCl_3_, δ, ppm): 2.45 (q, *J* = 6.9 Hz, 2H, C*H*_2_), 2.56 (t, *J* = 6.9 Hz, 4H, 2C*H*_2_), 3.48–3.73 (br, 2H, N*H*_2_), 6.40 (d, *J* = 8.47 Hz, 2H, aminophenyl-*H*), 6.58 (s, 1H, C=C*H*), 6.91 (s, 2H, benzene-fused-*H*), 6.98 (d, *J* = 8.47 Hz, 2H, aminophenyl-*H*). MS (NOBA, FAB > 0): 562 [M + H]^+^. Anal. calcd. for C_23_H_15_S_8_N: C, 49.16; H, 2.69; S, 45.65; found: C, 49.28; H, 2.84; S, 45.46.

### 3.7. Synthesis and Characterization of p-Dibenzylaminophenyl Benzene-Fused Bis Tetrathiafulvalene **12a**–**d**

K_2_CO_3_ (0.83 g, 6 mmol) was added to a stirred solution of 4-aminophenyl benzene-fused bis TTF **11a**–**d** (3 mmol) and benzyl bromide (0.71 mL, 6 mmol) in dimethylformamide (30 mL) under nitrogen. The resulting mixture was heated over an oil bath up to 120 °C and stirred for a further 2 h. The solvent was then removed under reduced pressure. Compound **12a**–**d** was obtained by column chromatography of the residue (silica gel, eluting with dichloromethane).

*p-*Dibenzylaminophenyl benzene-fused bis tetrathiafulvalene **12a**: Light yellow powder. Yield: 87%. M.p.: 195 °C. TLC: Rf = 0.81 (CH_2_Cl_2_). ^1^H NMR (400 MHz, CDCl_3_, δ, ppm): 4.67 (s, 4H, benzylamine-*CH*_2_), 6.37 (s, 2H, C*H*=C*H*), 6.54 (s, 1H, C=C*H*), 6.57 (d, *J* = 8.66 Hz, 2H, aminophenyl-*H*), 6.95 (s, 2H, benzene-fused-*H*), 7.15–7.25 (m, 10H, benzylamine-*H*), 7.35 (d, *J* = 8.66 Hz, 2H, aminophenyl-*H*). MS (NOBA, FAB > 0): 702 [M + H]^+^. Anal. Calcd for C_36_H_21_S_8_N: C, 58.16; H, 3.30; S, 36.53; found: C, 58.03; H, 3.18; S, 36.68.

*p-*Dibenzylaminophenyl benzene-fused bis tetrathiafulvalene **12b**: Wheat powder. Yield: 95%. M.p.: 208 °C. TLC: Rf = 0.76 (CH_2_Cl_2_). ^1^H NMR (400 MHz, CDCl_3_, δ, ppm): 1.95 (s, 6H, 2C*H*_3_), 4.65 (s, 4H, benzylamine-*CH*_2_), 6.52 (s, 1H, C=C*H*), 6.55 (d, *J* = 8.64 Hz, 2H, aminophenyl-*H*), 6.91 (s, 2H, benzene-fused-*H*), 7.13–7.27 (m, 10H, benzylamine-*H*), 7.31 (d, *J* = 8.64 Hz, 2H, aminophenyl-*H*). MS (NOBA, FAB > 0): 730 [M + H]^+^. Anal. calcd. for C_36_H_27_S_8_N: C, 59.22; H, 3.72; S, 35.13; found: C, 59.07; H, 3.58; S, 35.32.

*p-*Dibenzylaminophenyl benzene-fused bis tetrathiafulvalene **12c**: Yellow powder. Yield: 93%. M.p.: 213 °C. TLC: Rf = 0.66 (CH_2_Cl_2_). ^1^H NMR (400 MHz, CDCl_3_, δ, ppm): 4.66 (s, 4H, benzylamine-*CH*_2_), 6.53 (s, 1H, C=C*H*), 6.56 (d, *J* = 8.68 Hz, 2H, aminophenyl-*H*), 6.93 (s, 2H, benzene-fused-*H*), 7.14–7.25 (m, 14H, benzylamine-*H*, benzene-*H*), 7.35 (d, *J* = 8.68 Hz, 2H, aminophenyl-*H*). MS (NOBA, FAB > 0): 752 [M + H]^+^. Anal. calcd. for C_38_H_25_S_8_N: C, 60.68; H, 3.35; S, 34.10; found: C, 60.56; H, 3.25; S, 34.29.

*p-*Dibenzylaminophenyl benzene-fused bis tetrathiafulvalene **12d**: Gold powder. Yield: 85%. M.p.: 218 °C. TLC: Rf = 0.70 (CH_2_Cl_2_). ^1^H NMR (400 MHz, CDCl_3_, δ, ppm): 2.45 (q, *J* = 6.9 Hz, 2H, C*H*_2_), 2.56 (t, *J* = 6.9 Hz, 4H, 2C*H*_2_), 4.65 (s, 4H, benzylamine-*CH*_2_), 6.52 (s, 1H, C=C*H*), 6.55 (d, *J* = 8.65 Hz, 2H, aminophenyl-*H*), 6.91 (s, 2H, benzene-fused-*H*), 7.13–7.28 (m, 10H, benzylamine-*H*), 7.31 (d, *J* = 8.65 Hz, 2H, aminophenyl-*H*). MS (NOBA, FAB > 0): 742 [M + H]^+^. Anal. calcd. for C_37_H_27_S_8_N: C, 59.88; H, 3.66; S, 34.56; found: C, 60.13; H, 3.86; S, 34.26.

## 4. Conclusions

We herein describe the synthesis and the characterization of novel unsymmetrically benzene-fused bis tetrathiafulvalenes bearing alkyl chains at one end of the π-electron rich unit and different functional groups *p*-nitrophenyl, *p*-aminophenyl or *p*-dibenzylaminophenyl at the other extreme. Different routes and reaction conditions were explored to form these compounds.

The synthetic method requires the preparation of three new precursors the 4-(*p*-nitrophenyl)-1,3- dithiole-2-ylidenepiperidinium hexafluorophosphate **2**, 4-(*p*-nitrophenyl)-1,3-dithiole-2-selenone **3** and the 2-(4-(*p*-nitrophenyl)-1,3-dithiole-2-ylidene)-1,3,5,7-tetrathia-s-indacene-6-one **5**.

The electrochemical behavior of all donors was determined by cyclic voltammetry. Charge transfer complexes of the donors with TCNQ were prepared and the electrical conductivity of these materials was measured. Series of *p*-nitrophenyl benzene-fused bis tetrathiafulvalenes and *p*-aminophenyl benzene-fused bis tetrathiafulvalenes derivatives are conductors while series of *p*-dibenzylaminophenyl benzene-fused bis tetrathiafulvalenes are semi-conductors.

## Figures and Tables

**Table 1.** Potential of unsymmetrically benzene-fused bis tetrathiafulvalenes **10a**–**12d**.

Donor	*E*^1^_ox_ (mV)	*E*^2^_ox_ (mV)	*E*^3^_ox_ (mV)	Δ*E*_ox_ (mV)
**10a**	461	530	873	412
**10b**	459	528	870	411
**10c**	456	524	866	410
**10d**	457	525	867	410
**11a**	438	507	845	407
**11b**	436	504	842	406
**11c**	433	499	836	403
**11d**	434	501	838	404
**12a**	446	525	864	418
**12b**	445	522	861	416
**12c**	441	522	854	413
**12d**	443	523	858	415

**Table 2.** Energy level (eV) of the molecular orbitals for products **10a**–**12d**.

Compound	LUMO + 2	LUMO + 1	LUMO	HOMO	HOMO − 1	HOMO − 2	HOMO − 3
**10a**	−1.184	−1.460	−2.684	−4.901	−5.249	−6.459	−6.580
**10b**	−1.121	−1.414	−2.664	−4.807	−5.189	−6.330	−6.509
**10c**	−1.218	−1.471	−2.688	−4.977	−5.304	−6.460	−6.558
**10d**	−1.114	−1.421	−2.670	−4.782	−5.192	−6.289	−6.532
**11a**	−0.758	−0.912	−1.137	−4.597	−4.889	−5.636	−6.300
**11b**	−0.726	−0.868	−1.073	−4.542	−4.805	−5.602	−6.187
**11c**	−0.774	−0.940	−1.166	−4.631	−4.989	−5.651	−6.308
**11d**	−0.732	−0.863	−1.078	−4.538	−4.786	−5.604	−6.151
**12a**	−0.747	−0.900	−1.127	−4.564	−4.865	−5.416	−6.238
**12b**	−0.709	−0.859	−1.064	−4.507	−4.782	−5.385	−6.124
**12c**	−0.764	−0.927	−1.157	−4.596	−4.963	−5.432	−6.253
**12d**	−0.723	−0.851	−1.064	−4.516	−4.763	−5.389	−6.088

**Table 3.** Melting points and electrical conductivity of charge transfer complexes.

Complex	M.P (°C)	σRT (S cm^−1^)
**10a-TCNQ**	276	9.2 × 10^−2^
**10b-TCNQ**	281	4.8 × 10^−1^
**10c-TCNQ**	289	5.3 × 10^−1^
**10d-TCNQ**	294	8.7 × 10^−1^
**11a-TCNQ**	227	2.5 × 10^−2^
**11b-TCNQ**	231	1.7 × 10^−2^
**11c-TCNQ**	237	8.3 × 10^−1^
**11d-TCNQ**	240	7.6 × 10^−1^
**12a-TCNQ**	258	8.7 × 10^−6^
**12b-TCNQ**	263	5.3 × 10^−5^
**12c-TCNQ**	267	4.2 × 10^−4^
**12d-TCNQ**	272	1.8 × 10^−4^
